# Molecular and Structural Changes, and Skeletal Muscle Strength and Endurance in Chronic Obstructive Pulmonary Disease and Interstitial Lung Disease: Practical Applications of Assessment and Management

**DOI:** 10.3390/bioengineering13030329

**Published:** 2026-03-12

**Authors:** Nina Patel, Ahmet Baydur

**Affiliations:** 1 The USC Dornsife College of Letters Art and Sciences, University of Southern California, Los Angeles, CA 90089, USA; 2 Keck School of Medicine, University of Southern California, Los Angeles, CA 90012, USA

**Keywords:** chronic obstructive pulmonary disease, inflammation, interstitial lung disease, isokinetic and isometric dynamometry, muscle atrophy, muscle deconditioning, electromyography, oxidative stress, post-lung transplantation, pulmonary rehabilitation, skeletal muscle physiology

## Abstract

Chronic obstructive pulmonary disease, interstitial lung disease, and post-lung trans-plantation are often accompanied by skeletal muscle dysfunction that worsens the quality of life. Such physiological changes are driven by physical inactivity, systemic inflammation, oxidative stress, anabolic and hormonal resistance, and medication effects. Structural changes include impaired capillarization, fiber-type shifts (slow-to-fast in limb muscle and fast-to-slow in respiratory muscles), mitochondrial dysfunction, reduced oxidative capacity, and early lactate accumulation. Electromyography and dynamometry, both isokinetic and isometric, quantify neuromuscular drive through measuring strength, power, and endurance and are associated with functional outcomes (6-min walk, sit-to-stand, stair climbing tests). Pulmonary rehabilitation (PR) improves neuromuscular efficiency, dyspnea, exercise tolerance, and quality of life by combining resistance, endurance, and eccentric training. The effects of PR generally plateau at three months, emphasizing the need for maintenance and the personalization of rehabilitation plans. While nutritional optimization is important, supplements have shown little benefit. Future priorities include defining EMG/dynamometry thresholds to allow standardized routine testing for comparable benchmarks and more precise PR protocols. Future research targeting mitochondrial remodeling, inflammatory signaling, and anabolic resistance offer potential pathways for preventing and reversing muscle wasting.

## 1. Introduction: Physiological Changes in Skeletal Muscle in ILD, COPD, and Post-Transplant Patients

Chronic obstructive pulmonary disease (COPD) and interstitial lung disease (ILD) are debilitating respiratory conditions characterized by significant skeletal muscle dysfunction, profoundly impacting patient disability, morbidity, and general quality of life (Table 1) [1]. Systemic molecular and physiological changes affect the structure and function of skeletal muscles [2]. Reductions in muscle mass, strength, and endurance compromise exercise tolerance [3,4,5]. Other contributing factors include hormonal imbalances and oxidative stress [2,5]. Systemic inflammation inhibits protein synthesis, promotes protein muscle degradation, and contributes to muscle wasting [4]. Growth hormone, while maintaining its plasma concentration, exhibits decreased interaction with insulin-like growth factor, impairing proteostasis and decreasing muscle mass [6]. Pro-inflammatory cytokines such as tumor necrosis factor-alpha (TNF-α) and interleukin-6 (IL-6) are associated with the pathogenesis of muscle atrophy. Increased levels of catabolic hormones such as cortisol, and decreased levels of anabolic hormones such as testosterone, enhance muscle weakness and loss [6]. Structural alterations in skeletal muscle include a decrease in fatigue-resistant type 1 fiber size, which is important for endurance activities [5]. We review herein the background and recent developments in the field, including skeletal muscle structural and molecular changes occurring with chronic lung disease, methods of evaluating muscle function, and the benefits of pulmonary rehabilitation (PR) in the prevention and reversibility of such changes.

### 1.1. Methods of Literature Review

For this review, we conducted a non-systematic literature search using the PubMed, Scopus, and Web of Science databases. We also searched the Cochrane Central Register of Controlled Trials via the Cochrane Register of Studies, MEDLINE (OvidSP), Embase (OvidSP), and the Physiotherapy Evidence Database (PEDro). Search terms prioritized key mechanistic and clinical studies. We included “skeletal muscle weakness”, “fatigue”, “skeletal muscle fiber types”, “electromyography”, “dynamometry”, “chronic obstructive pulmonary disease”, “interstitial lung disease”, “lung transplantation”, “inflammation”, “oxidative metabolism”, “pulmonary rehabilitation”, “quality of life”, and “respiratory function”. We screened and evaluated relevant studies and reviews in English published from 1977 through 2025. The selection of references was based on their relevance to the pathophysiology, assessment, and management of limb muscle weakness, with new information on laboratory studies, key observational studies, and systematic reviews. We excluded discussions of skeletal muscle changes occurring with neuromuscular disease, as they do not directly result from lung disease, and the topic is covered extensively in the neuromuscular literature. Given the narrative nature of this review, we did not perform a formal risk of bias assessment, independent duplicate screening, or meta-analysis.

### 1.2. Skeletal Muscle Dysfunction in COPD: Molecular Mechanisms

**Figure 1:**
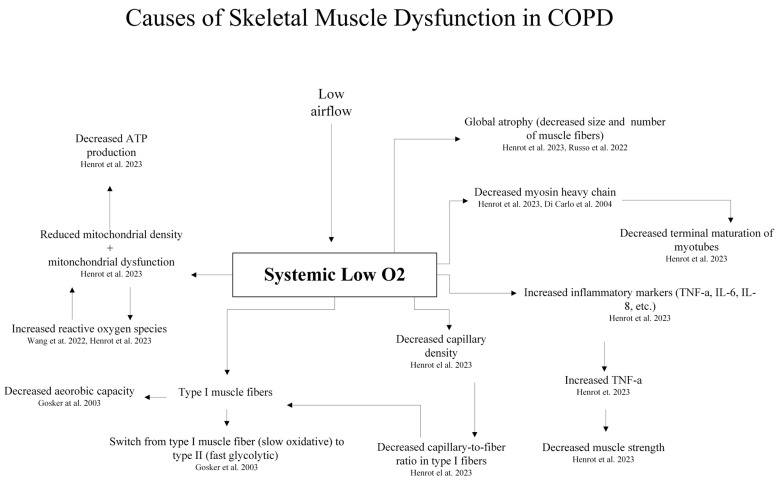
Skeletal muscle dysfunction in COPD arises from systemic inflammation, corticosteroid use, hypoxemia, oxidative stress, physical inactivity, nutritional depletion, and comorbidities. This leads to impaired mitochondria function, fiber-type shifts, muscle atrophy, and ultimately reduced strength and endurance that contributes to dyspnea and exercise intolerance [56,58,59,60,61].

Skeletal muscle wasting in COPD is attributed to physical inactivity resulting from expiratory flow limitation (EFL) and dynamic air trapping leading to exertional dyspnea and loss of motivation to exercise [1]. Studies of skeletal muscle dysfunction in COPD have primarily focused on the quadriceps [56]. EFL decreases systemic oxygenation, impairing muscle aerobic capacity, force generation, and endurance. Muscle fibers switch from a slow oxidative to a fast glycolytic type and exhibit a decrease in cross-sectional area (CSA) resulting in global atrophy (Figure 1) [56,57,58]. Capillarization is impaired, with decreased capillary density and capillary-to-fiber ratio in type 1 muscle fibers, altering oxidative capacity [56].

Patients exhibit elevated serum inflammatory markers such as tumor necrosis factor (TNF-α), interleukin-6 (IL-6), and interleukin-8 (IL-8) (Figure 1), which negatively associate with lean muscle mass and reduced strength in cachectic patients. TNF-α may play a role in promoting inflammation and catabolism through the NF-κB pathways as well as anabolic effects such as promoting satellite cell differentiation [56]. Yet, therapies targeting TNF-α have not consistently improved dyspnea or COPD exacerbation rates, indicating that it is not the key driver of muscle dysfunction [56]. Similarly, while IL-6 and IL-8 are related to catabolic effects, the transcriptional levels of these interleukins are generally unchanged in COPD patients [56]. Patients with COPD exhibit reduced muscle phosphocreatine (PCr) and ATP, but increased lactate and inorganic phosphate [7,8,57]. Blood lactate levels increase rapidly with minimal exertion [9]. In many respects, such changes parallel events occurring in cancer and sepsis [7,9,56,57,58].

Hypercapnia and hypoxia induce reductions in mitochondrial density, muscle fiber size, and muscle mass (Figure 1) [59]. Members of the hypoxia inducible factor 1 (HIF-1) family regulate the expression of several genes involved in adaptive processes that facilitate cell survival under hypoxic conditions [62]. Several different HIF-1- and chromatin-associated co-regulators play important roles in the transcriptional activity of HIF-1, and in the selection of binding sites, promoters, and target genes [60,62]. Hypoxia decreases terminal maturation of myotubes through decreasing myosin heavy chain [56,62]. Mitochondrial dysfunction manifests as reduced membrane potential, deficiency in important mitochondrial proteins, impaired oxidative phosphorylation, and increased mitochondrial reactive oxygen species [56,60]. Smoking, hypoxia, and physical inactivity are exacerbating factors (Figure 1) [60]. Gender plays a role in muscle dysfunction with greater weakness and impairment in women than in men. Female COPD patients possess smaller type II fiber CSAs compared to men, and higher levels of pro-inflammatory cytokines such as TNF-α and IL-8 [10,11].

The maintenance of muscle mass is maintained by signaling pathways that regulate protein synthesis and degradation, including the transforming growth factor beta (TGFβ) receptor superfamily, the ubiquitin–proteasome axis, calcium-dependent proteolysis, and autophagy cascades [63,64]. While basic studies in muscle atrophy involve animal and cell models of cancer [63,64], the same molecular mechanisms are likely active in the cachexia of chronic inflammatory conditions, as in COPD and ILD.

The sedentary lifestyle associated with advanced chronic lung disease suppresses autophagy activity [65]. Exercise is an autophagy inducer and is thought to contribute to the beneficial effects of exercise. Based on an acute exercise model in mice, Li et al. [65] identified lactate as a positive regulator of autophagy in skeletal muscle. Their mechanistic study showed that lactate enhances autophagy by inactivating the mammalian target of rapamycin complex 1 (mTORC1) through promoting mTOR lactylation. mTOR could be lactylated at myocyte lysine 921 (K921), which inactivated mTORcomplex1 (mTORC1), thereby linking lactate signaling to mTORC1-controlled autophagy during exercise. Their findings suggest a mechanism for explaining exercise-induced muscle adaptation, and a potential treatment strategy for muscle disuse and atrophy in individuals with chronic respiratory disease.

Mitochondrial dysfunction contributes to skeletal muscle atrophy, playing an important role in energy production, metabolic flexibility, controlling redox homeostasis, and regulation of apoptosis. Signaling pathways involved in skeletal muscle atrophy include the ubiquitin–proteasome system, autophagy, mitophagy, mitochondrial fission–fusion, and mitochondrial biogenesis [66].

Non-coding RNAs (ncRNAs) contribute to muscle dysfunction and muscle loss in patients with COPD [67,68,69,70]. The plasma levels of several myo-specific c-miRNAs are increased in patients with COPD and are possibly closely related to muscle turnover [59,67,68,69,70]. A reduction in skeletal muscle mass is associated with metabolic disorders such as insulin resistance and is a risk factor for cardiovascular diseases [59,68,69,70], which is also likely in patients who take inhaled glucocorticoids for COPD [71].

The myriad pathways contributing to muscle disuse and atrophy are frequently the consequence of environmental exposures as well as the mechanical stresses under which such patients labor, but at the same time they offer the potential for ameliorating and even reversing the associated physical impairment (discussed below).

### 1.3. Skeletal Muscle Dysfunction in ILD

**Figure 2:**
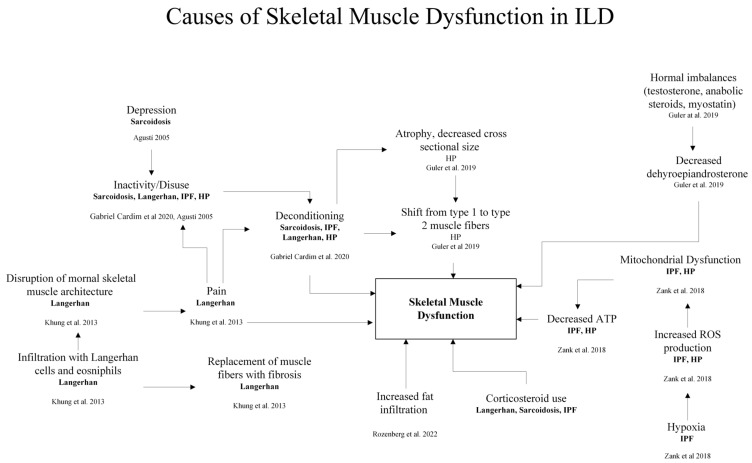
Causes of skeletal muscle dysfunction in ILD. Hypoxia, hormonal imbalances, medication use, mitochondrial dysfunction, mental health, pain, and disuse all contribute the physiological changes seen in ILD patients, such as muscle atrophy, fiber-type shift, and muscle infiltration that leads to skeletal muscle dysfunction [13,35,72,73,74,75].

As in COPD, deconditioning in ILD develops with inactivity and disuse as patients try to avoid dyspnea. In addition, changes in growth and sex hormones alter muscle function and mass. Patients with idiopathic pulmonary fibrosis (IPF) exhibit decreased levels of dehydroepiandrosterone (DHEA); anabolic steroids, myostatin, and testosterone have been related to disease-related wasting as in COPD patients (Figure 2) [12,35].

Fat infiltration of lower extremity muscles impairs mobility and muscle performance, particularly in older individuals (Figure 2) [13]. Similar to COPD, skeletal muscle atrophy occurs more in lower than in upper limbs [14]. There is greater muscle fat in patients experiencing functional impairment. Yet upper limb muscle dysfunction and decreased physical performance are not related to body fat percentage or muscle mass. The existence of alternative pathways in which limb muscle function and performance are affected by ILD [35], highlights a need for future study to clarify which alternate pathways that affect this process.

Oxygenation levels in the quadriceps of patients with ILD are decreased compared to intercostal muscles at rest, which is related to reduced blood flow to the lower extremities [15]. Yet, in a near-infrared spectroscopy (NIRS) study (a non-invasive technique used to determine the relative oxygen saturation of myoglobin and hemoglobin) comparing oxygen use in 30 healthy individuals and 30 patients with mild ILD. Pehlivan et al. [15] found that oxygenation of the exercising limb was similar in both groups. Instead, they proposed that the increase in the work of breathing diverted blood away from lower extremities, resulting in exercise intolerance [15].

### 1.4. Skeletal Muscle Dysfunction in Post-Lung Transplant Patients

**Figure 3:**
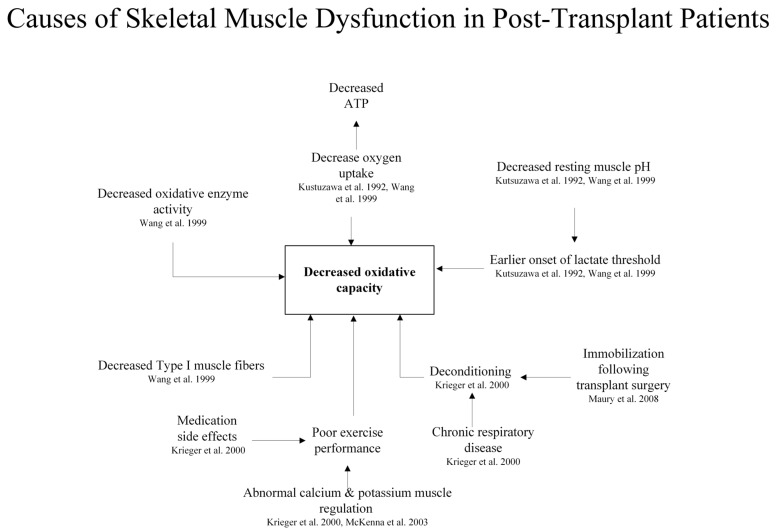
Causes of skeletal muscle dysfunction in post-transplant patients. Skeletal muscle dysfunction after solid organ transplantation arises due to the use of immunosuppressive therapy, systemic inflammation, electrolyte imbalances, and prolonged pre-transplant deconditioning. These factors contribute to mitochondrial dysfunction, muscle atrophy, fiber-type shifting, and oxidative stress. This ultimately impairs strength, endurance, and overall functional recovery in post-transplant patients [17,18,19,44,76].

Following lung transplantation, patient outcomes are associated with skeletal muscle dysfunction. In both single and double lung transplant patients, work capacity and peak oxygen uptake (VO_2_max) remain decreased despite exercise training [76]. NIRS shows altered venous and oxygen content and increased cardiac output (CO) before and after transplantation (Figure 3) [76]. Peak VO_2_ decreased in post-transplant patients 5 to 38 months after transplantation, although impaired ventilation, anemia, and arterial oxygen saturation do not account for these changes [16]. Nuclear magnetic resonance spectroscopy (NMR) analysis during quadriceps exercise exhibits a decrease in resting muscle pH (7.07 in post-transplant patients vs. 7.12 in healthy volunteers), an earlier onset of lactate threshold, and decreases in oxygen uptake and oxidative capacity (Figure 3) [16,17,18], ascribed to immunosuppressant toxicity [76] (Figure 3).

Skeletal muscle dysfunction immediately following transplantation is attributed to prolonged immobility in intensive care (Figure 3) [44]. Poor exercise performance relates to disorders of muscle calcium and potassium regulation: While muscle sarcoplasmic reticulum calcium is downregulated with decreased calcium release and calcium uptake, Sodium–Potassium Adenosine Triphosphatase (Na^+^-K^+^-ATPase) activity is increased by 31% [19], suggesting a dissociation in the expression of sarcoplasmic reticulum function and myosin ATPase (Figure 3) [19,76]. Exercise intolerance in post-transplant patients is associated with slow-to-fast muscle fiber conversion, reduced ATP content, and de-creased oxidative enzyme activity [17]. Immunosuppressants, such as cyclosporin A, inhibit calcineurin-activated pathways, also leading to slow-to-fast muscle fiber conversion [19].

## 2. Methods of Evaluating Muscle Dysfunction

### 2.1. Electromyography (EMG) Analysis for Quantifying Skeletal Muscle Dysfunction: Technical Challenges

Electromyography (EMG) offers valuable insights into neuromuscular function with its ability to assess changes in muscle activation, muscle fiber recruitment, and neuromuscular transmission by recording the frequency and amplitude of electrical signals during muscle activation [77,78,79]. The decreased EMG amplitude recorded during maximal contractile effort indicates muscle weakness or impaired neuromuscular drive [80].

The surface myoelectric signal (MES) has been used as an input to controllers for powered prostheses for many years. Amongst the concerns of recording EMG signals have been differences between surface and intramuscular needle electrodes in signal detection. A potential disadvantage of MES is the loss of more global information contained in the surface MES. Hargrove and colleagues [81] studied 12 normally limbed subjects by placing four equally spaced surface electrodes to the circumference of the forearm. They compared the classification accuracy of six pattern recognition-based myoelectric controllers using multi-channel surface MES as inputs to the same controllers which used multi-channel intramuscular MES as inputs, with both sets of signals collected simultaneously for 10 different classes of isometric contraction. The investigators found that information obtained from either surface or intramuscular sensors provided excellent classification accuracy; both measurement techniques yielded classification accuracies between 95% and 99%.

The normalization of signals has been another challenge, with different approaches utilized to address it. Shin et al. [78] used surface EMG electrodes set by Surface Electromyography for the Non-invasive Assessment of Muscles (SENIAMs) [82]. Subjects performed two 5-s-long maximum voluntary isometric contractions (MVICs). The root mean square (RMS) value of the rectus femoris muscle divided by the MVIC was used to analyze the normalized RMS in the ramp exercise test. Gephine et al. [83] compared quadriceps oxygenation and surface EMG (sEMG) responses during 1-min sit-to-stand (1STS) tests in 14 individuals with severe COPD and 12 controls. sEMG signals were normalized for each sit-to-stand (STS) (one cycle) test using electrogoniometer signals during the 1STS.

Shifts to a lower frequency indicate reduce motor unit firing velocity and fatigue-related slowing of muscle fiber conduction velocity [84,85]. Fourier analysis of force-frequency changes occurring during muscle fatigue converts the EMG signal from the time domain into the frequency domain demonstrating slowing muscle fiber conduction during fatigue [86]. In experimental studies, the median frequency consistently decreases more than muscle fiber conduction velocity (MFCV). Lowery et al. [84] compared a new estimate of EMG frequency compression, the spectral compression estimate (SCE), with the median frequency of the EMG power spectrum, the median frequency of the EMG amplitude spectrum and MFCV measured during sustained isometric, fatiguing contractions of the brachioradialis muscle at 30, 50, and 80% maximum voluntary contraction. They found that the SCE provided a better estimate of the observed changes in MFCV than the median frequency of either the EMG power spectrum or EMG amplitude spectrum.

The net force exerted by muscle depends on the magnitude and timing of motor unit activity, the contractile properties of the activated muscle fibers, and the mechanical characteristics of the connective tissues transmitting the forces to the limb [87]. Limitations in interpreting motor unit activity from EMG recordings include the consequence of dissociation between motor unit characteristics and muscle fiber types. For example, the number of motor unit action potentials may not directly relate to the number of muscle fiber action potentials that contribute to an EMG signal [87]. As such, the EMG signal amplitude is not a direct index of the neural drive to muscle, especially during fatiguing contractions. Still, the signal amplitude provides a useful approximation of the amplitude of the neural drive to muscle during some controlled conditions.

COPD patients have decreased respiratory neuromuscular efficiency (reduced tidal volume with respect to diaphragm EMG output) with moderate exercise intensity and decreased peripheral neuromuscular efficiency (relationship between power output and vastus lateralis EMG) with just light exercise intensity [20]. Calatayud et al. [21] investigated the acute symptomatic and neuromuscular responses to increased elastic resistance exercise in 14 patients (9 male) with COPD. Neuromuscular activity was recorded during knee extensions against graded elastic resistances using sEMG for separate quadriceps muscles, together with the rate of perceived exertion, perceived quadriceps fatigue, dyspnea, oxygen saturation, and heart rate (HR). In most cases, at least a two-level resistance increase was needed to obtain a significant normalized root-mean-square increase during knee extensions. In some cases, EMG may not reflect minute changes in muscle function, such as the degree of activation or decreased oxidative capacity [36].

In summary, sEMG recording records the electrical activation of limb muscles while supporting clinical assessment and management of patients with chronic respiratory disease [78]. Standardizing the acquisition and presentation of limb muscle sEMG outcomes can be a challenge when reporting details of the applied hardware and software methods. Because sEMG is dynamic, guidelines should be established on how best to assess the reliability of recordings, thereby establishing the optimum variables and cutoff values that indicate relevant clinical changes. In doing so, sEMG can facilitate formulating a personalized training program.

### 2.2. Isokinetic vs. Isometric Dynamometry for Quantifying Skeletal Muscle Dysfunction: Strengths, Limitations

- Isokinetic dynamometry:

Isokinetic dynamometry is often regarded as the “gold standard” for measuring muscle strength and power in both orthopedic and neurological patient populations [38]. It assesses muscle strength by measuring maximal torque or force generated during a dynamic contraction at a constant angular velocity. Compared to isometric dynamometry, it provides a more comprehensive evaluation of endurance and dynamic strength across a scope of motions and velocities [36,37,38]. The review by Bohannon et al. [37] found that, in general, reliability coefficients reported for isokinetic measurements in COPD were high yet were limited to test–retest reliability and derived from studies with fewer than 15 participants. These limitations impaired the ability to establish meaningful goals and interpret changes in patient performance over time.

Typically, isokinetic measurements are performed at fixed angular velocities of 60° to 90° [88]. Evaluation of dynamic muscle strength has relatively high reliability, while evaluation of static muscle strength is of moderate to high reliability [39,88]. Saey et al. [39] found that a 30-maximum repetition isokinetic muscle endurance test could be performed in a reliable fashion in sedentary patients with moderate to severe COPD and similar body composition, particularly at 90° per second. While isokinetic peak torque (Tq) and total muscle work were reliable at angular velocities of 90° and 180° per second, test–retest measurements of work slope and fatigue index (FI) were only reproducible at 90° per second. Meanwhile, changes in dyspnea and leg fatigue perception scores failed to achieve acceptable test–retest reproducibility, regardless of the velocity applied. Saey et al. [39] recommended the development of endurance muscle testing in patients with COPD, an area for which no standardized guidelines are available.

Isokinetic dynamometry has been used to determine muscle fiber distribution as slower velocities indicate type I fibers and faster velocities emphasize type II fibers. In a murine study, Kupa et al. [89] compared surface-detected EMG median frequency and conduction velocity (CV) variables with histochemical characterization of muscle fiber-type composition and CSA. EMG signals were recorded during tetanic contractions from different muscles. Muscles with faster glycolytic fibers exhibited higher initial values and more reduction in MF and CV during contraction. Fiber-type composition could be predicted based on MF parameters. Nevertheless, multiple factors other than fiber composition influence fiber type. Moreno-Justicia et al. [90] applied transcriptomic and proteomic workflows to single myofibers from human vastus lateralis in patients with nemaline myopathy. They identified metabolic, ribosomal, and cell junction proteins, as well as myosin heavy chain isoforms, as sources of variation between myofibers. They did not, however, study individuals with chronic lung disease.

Amaral et al. [91] described the contribution of mechanical and physiological factors to torque generation as being additionally represented by the maximum work repetition number, work fatigue percentage, and angle of peak torque. They suggested that maximum work should be employed to characterize torque generation capacity assessments. Alt et al. [92] evaluated the effects of angular velocity and training status on dynamic control equilibrium, a modification of the quotient of maximal eccentric hamstring and maximal concentric quadriceps moments in 58 trained and 58 untrained male participants. Higher DRCe moments and angles were associated with an increased capacity to resist high eccentric knee flexor moments, particularly during rapid knee extensions. Employing such an approach has clinical potential in patients with chronic lung disease to compare changes in limb muscle imbalance before and after pulmonary rehabilitation. As can be seen, the variability of which functional indices are assessed calls for standardization in clinical application.

In summary, patients with COPD and ILD exhibit reductions in maximum torque and power and increased fatiguability [3,22,23,24,45]. From a clinical standpoint, isokinetic quadriceps strength associates with functional outcomes such as stair climbing power, STS, timed-up-and-go, gait performance, and six-minute walk distance [24,25,40]. Addition-ally, isokinetic testing exhibits gender-related differences in thigh muscle strength for COPD patients, with females showing an increased tendency for decreased thigh muscle strength [93]. Increases in strength following neuromuscular electrical stimulation, whole body exercises, and resistance training consistently follow interventions [23,46]. Additional research is required to create guidelines for standardized metrics which should enable isokinetic testing to be increasingly clinically applicable [39].

b Isometric dynamometry:

Isometric dynamometry measures the maximum force generated against resistance at a fixed muscle length and joint angle. It is typically conducted with hydraulic resistance devices or force transducer platforms adapted to weight machines [39]. While more accessible for clinical settings, it provides limited insight into dynamic muscle function or endurance [41]. Testing at a 90° knee angle is recommended for quadriceps strength assessment [26,47,48]. Handheld dynamometry (isometric) is another reliable method of measuring elbow flexor and knee extensor strength for patients with ILD [48].

## 3. Reversing Physiological Changes Through Pulmonary Rehabilitation (PR)

Pulmonary rehabilitation (PR) is a multi-faceted intervention that utilizes exercise training as a central therapeutic modality. Individually tailored exercise programs encompass both resistance and endurance training. They improve quality of life and functional capacity in both COPD and ILD [1,49,50,51,94]. Dowman et al. [49] found that participants with idiopathic pulmonary fibrosis (IPF) exhibited improvements in six-minute walk test results, maximum exercise capacity, dyspnea, and health-related quality of life. The increase in the 6MWT was similar among participants with IPF (37 m) and other forms of ILD (40 m in all individuals) and exceeded the minimal clinically significant difference for the 6MWT in people with IPF (29 m to 34 m) [95].

Exercise increases energy demand with the production of myokines and metabolic intermediates such as lactate. This leads to benefits including improved insulin sensitivity, which is potentially important in COPD patients who receive glucocorticoids for disease control. Mechanical loading during exercise attenuates a loss of muscle mass and function [96]. Mechanotransducers and other stress-signaling compounds promote skeletal muscle adaptation in response to exercise [97,98].

While measuring quadriceps muscular strength is recommended in COPD during a pulmonary rehabilitation program, the instruments for assessing quadriceps maximal voluntary contraction (QMVC), and the clinical relevance of the results for a given patient need further refining. Vaidya et al. [99] evaluated the minimal important difference (MID) of QMVC using fixed handheld dynamometry in 157 COPD patients (mean FEV1 [forced expiratory volume] 47% predicted). They found that simple QMVC evaluation using a fixed dynamometer is reliable for depicting improvement of quadriceps muscle strength by the end of a PR program. Similarly, Bui et al. [41] aimed to validate the test–retest reliability of an isometric maximal voluntary contraction (iMVC) testing protocol in 31 individuals with COPD (mean FEV1 52% predicted) using a commercially available handheld dynamometer on two separate days. They found an intra-class correlation coefficient of 0.96, a mean variation of 0.7% between the two visits, and a mean of the difference of 1.6% between visits, concluding that standardized handheld quadriceps dynamometry is a reliable tool to assess iMVC in people with COPD.

Changes in contractile activity induce remodeling of biochemical, metabolic, and force-generating properties [100]. The transcriptional response to acute exercise reverts to baseline within hours to days, with significant variations amongst individual genes, highlighting the importance of patients with chronic lung disease maintaining a consistent program during pulmonary rehabilitation. Various factors modify the response of skeletal muscle to exercise, including training strategies, age, or sex. Resistance exercise (RE) is characterized by high intensity and short duration, and promotes the synthesis of contractile and structural proteins, leading to muscle hypertrophy [101,102]. In summary, numerous signals, sensors, regulators, and effectors are involved in these adaptive processes. Mechanisms underlying signal integration, output coordination, and other complex traits of muscle adaptation need further elucidation.

In a small pilot study, Grandio et al. [52] found that an 8-week PR program resulted in less quadriceps stimulation to generate the same torque as pre-PR (Table 1). These changes aligned with an improvement in quality-of-life scores and survival. Patients with pulmonary fibrosis who exhibited increased physical performance during PR exhibit improved survival [53]. Both resistance and endurance training improve quadriceps muscle function in patients with COPD [54]. Endurance, but not resistance training, resulted in a decrease in the proportion of glycolytic type IIa muscle fibers and phosphofructokinase content in muscle protein, and an increase in citrate synthase. Functional performance declined after 3 months of PR and was associated with reversal of such changes in muscle properties. Some pulmonary rehabilitation programs incorporate eccentric resistance training to counteract this decline [55]. Vaes et al. [27] found a positive relationship between quadriceps endurance and strength and exercise performance in COPD patients by measuring peak oxygen use (VO_2_) and walking distance in patients (Table 1). While both factors contributed to exercise intolerance, endurance was a stronger predictor of exercise capacity than quadriceps strength. Additionally, quadriceps function was more strongly related to peak VO_2_ than 6MWT [27].

While respiratory and limb muscle dysfunction exhibit similar alterations, the diaphragm exhibits greater fatigue resistance with a predominance of slow-twitch muscle fibers in contrast to limb muscles which possess relatively more glycolytic type 2 fibers. Both muscle groups are subject to oxidative and mitochondrial stress, the activation of proteolytic enzymes, and increased inflammatory mediators [1,16,28,42,56,103,104,105]. Respiratory muscles adapt with fast-to-slow fiber shifts with greater oxidative capacity, while lower limb muscles adapt with atrophy, fat infiltration, and slow-to-fast muscle fiber shifts [29,30,31,32,33,34,105]. As such, factors that improve ventilation and limb muscle function must be layered together. Muscle injury can be prevented through the appropriate use of bronchodilation, oxygen supplementation, and assisted ventilation; muscle regeneration can be facilitated through endurance training [106,107].

Many PR programs incorporate nutritional counseling to address muscle wasting and malnutrition. Nutritional supplementation focuses on optimizing protein intake and addresses nutrient deficiencies to facilitate muscle repair and growth [108]. While supplements such as creatine improve exercise capacity in healthy people, they have not shown clear improvements in COPD patients [109]. Supplements like L-carnitine, involved in fatty acid oxidation in mitochondria, benefit athletes but show only modest improvements in COPD patients. Anabolic drugs, including testosterone, oxandrolone, selective androgenic receptor modulators, or growth hormone, have positive effects on muscle mass, but no clear functional benefits [108]. Other supplements have shown promise [109,110], but clinical trials are needed for confirmation.

## 4. Quality and Strength of Review and Meta-Analysis Papers Discussed

The analytical rigor utilized in the meta-analyses and systematic reviews discussed herein varies. In general, limitations amongst systematic reviews include differences amongst training protocols, outcome definitions, small sample sizes, and an incomplete blinding of outcome assessments (Table 1). For example, the systematic review of van der Woude discussing isokinetic dynamometry in the evaluation of muscle strength and power was comprehensive and utilized the PubMed, Cumulative Index of Nursing and Al-lied Health (CINAHL), and Embase electronic databases. First, they assessed the methodological quality of the studies according to the Consensus-Based Standards for the Selection of Health Measurement Instruments guidelines (COSMIN) (Van Der Woude et al., 2022) [38]. Next, the authors determined the methodological quality and combined the quality of measurement properties of the studies to obtain a best-evidence synthesis. Their study was limited, however, to patients with neuromuscular disorders (NMDs) and did not include patients with chronic lung disease.

Bohannon [37] conducted a search of isokinetic testing procedures in older adults with COPD, identifying 27 suitable articles. Only one bibliographic database (PubMed) of note was searched. A wider search may have provided more evidence for the conclusions presented and allowed for a meta-analysis of some variables and for a quality grading of the included articles. In addition, only a single examiner (the author) conducted the literature search. The review of pulmonary rehabilitation in individuals with ILD by Dowman et al. [49] incorporated a pooled quantitative analysis of clinically homogeneous trials and a fixed-effect or random effects model depending on an assessment of heterogeneity using the Chi2 test and the I2 statistic. They utilized multiple search engines, identifying 21 eligible studies comparing pulmonary rehabilitation (yes or no) or sham control among people with a variety of ILDs.

The meta-analysis of Li et al. [51] pooled data using mean and standardized mean differences (SMDs) with subgroup analyses based on the type of training modality (Table 1 in ref. [51]). A random-effects model was used for analysis. The authors used the percentage of variation (I2 statistic) to assess heterogeneity across studies. Sensitivity analyses were conducted to assess the source of heterogeneity based on the intervention program characteristics of the participants when subgroup analysis could not determine the source of heterogeneity. The quality of methodology of randomized control trials was assessed using the physiotherapy evidence database (PEDro) scale, which includes 11 items with 10 scores with higher scores indicating better quality.

By contrast, the review of diagnostic and therapeutic approaches for limb and respiratory dysfunction in COPD by Barrio and Gea et al. [4,6] does not describe selection methods nor provide a quality assessment of the studies reviewed. A targeted literature review of the molecular and structural changes in the skeletal muscle of COPD patients by the same group [111] exhibits the same limitations. Jaitovich et al. [42] reviewed the mechanistic aspects of muscle dysfunction in patients with COPD, utilizing more than 200 references to describe mitochondrial metabolic dysfunction. The authors integrated biomolecular and metabolic aspects of muscle wasting with various limb training and PR programs employed to reverse muscle weakness and improve quality of life. There was no discussion of between-study heterogeneity or a comparison of molecular techniques amongst different in vitro studies.

Gosker et al. [31] screened electronic databases to find studies in which quadriceps fiber-type proportions were determined in patients with COPD. PubMed, EMBASE, and the Cochrane Library were searched using various terms relevant to muscle fiber types, myosin heavy chains, and COPD. Online abstracts of relevant conferences were also screened from the American Thoracic and European Respiratory Societies. From the selected reports, fiber-type composition, and respiratory function data were extracted and age-matched with healthy control groups of the selected COPD-related papers.

An excellent review of the state of the field by Maltais et al. [1] provides an update of the current scientific and clinical knowledge on the topic and provides guidance for future research directions. A librarian was consulted to perform a search of the literature using PubMed, Embase, and CINAHL, incorporating several terms relevant to chronic lung disease and muscle strength, wasting, and oxidative stress. The authors also ensured that the methodological process was consistent with the approved methodology of the American Thoracic Society documents development and implementation committee.

A number of small interventional pilot studies should be interpreted cautiously be-cause of limited power and potential type II error [18,52,54,55].

## 5. Future Considerations for Investigation

Further investigation on mitochondrial remodeling and mechanisms linked to hypoxia, oxidative stress, and mitochondrial biogenesis failure would be useful to identify antioxidant or mitochondrial-targeted therapies. There is a need to clarify anabolic resistance and cortisol/testosterone imbalances as they relate to sex to inform hormone-based trials. Electrical stimulation and pharmacologic modulators to promote type I fiber regeneration should be further explored.

Studies involving EMG and isokinetic/isometric monitoring would be key in defining signal thresholds as they relate to PR. An area of interest would be to compare the ratio of quadriceps peak EMG signal to the torque produced during knee extensions during isokinetic dynamometry in patients with COPD and those with restrictive disorders [52]. One would expect a higher ratio in COPD because of the greater diaphragm force generation required to overcome air trapping and the shorter resting length of diaphragm fibers. As respiratory pressures are related to lung volume, one would also expect a high ratio in ILD patients.

Magnetic resonance imaging (MRI) or NIRS can be paired with EMG and dynamometry to visualize intramuscular oxygen concentration and microvascular changes. Assessment of PR components may identify methods to sustain PR benefits beyond three months. Studies of dietary supplementation to enhance strength, endurance, and survival outcomes would facilitate the individual personalization and tailoring of programs.

Shear wave ultrasound elastography (UE) has the potential to assess muscle dysfunction in ILD and COPD by measuring muscle stiffness and elasticity. Strain elastography (SE), acoustic radiation force impulse (ARFI) technology, and shear wave elastography (SWE) are the three main UE methods [111,112,113,114]. UE should be considered to assess structural changes in limb muscle before and after PR.

The study of environmental factors such as smoking, vaping, and exposure to particulate matter may reveal their effects on ncRNAs synthesis. Future research can be directed to (1) determine whether ncRNAs can become new therapeutic targets or diagnostic markers; (2) assess whether ncRNAs provide a means for optimizing rehabilitative management of muscular atrophy in individuals with chronic lung disease; and (3) generate the development of novel and effective nutritional supplements, drugs, and targeted therapy in such patients [67].

Clarifying molecular pathways leads to new drug targets to prevent muscle wasting. ncRNAs have the potential to become new biomarkers as diagnostic tools and therapeutic targets for muscle atrophy in patients with COPD and ILD, including those with CTD-ILD. Identifying novel ncRNA species, investigating their targets and specific mechanisms of action can provide information regarding the regulatory roles of ncRNAs in muscle atrophy, which may allow drug development targeting the expression and activity of disease-related ncRNAs [67,68,69,70].

An intriguing avenue for investigation is the potential use of the mTOR inhibitor FK506 in the prevention and reversal of muscle atrophy associated with chronic wasting diseases [115], including COPD and ILD. In cachectic tumor-bearing mice, FK506 pre-vents muscle and body weight loss and protects from neuromuscular junction alteration by inhibiting the intracellular FK506 binding protein 12 (FKBP12), an immunophilin that limits and prevents uncontrolled activation of bone morphogenetic protein (BMP) signaling by binding the cytoplasmic domain of the type I BMP receptor [115]. To further elucidate the impact of FK506 treatment on COPD or ILD-induced cachexia (as it is applied in cancer models), employing the agent in animal models of emphysema or pulmonary fibrosis should be considered as a next step, followed by human trials (if animal studies are promising).

Given that structural changes occurring with chronic lung disease mimic events in aging (4 Barreiro, Gea), such as telomere attrition, inflammation, and mitochondrial dysfunction [116], stem cells may serve as a replacement for atrophic myocytes. Muscle stem cells are needed for regenerating skeletal muscle under resting conditions, during which they remain quiescent. During regeneration after injury (mitochondrial and oxidative damage), they become activated, begin to proliferate, and can differentiate into muscle fibers or replenish the stem cell pool [117]. In a murine model, Kang et al. [118] recently reported an increase in the expression of the tumor suppressor gene N-myc downregulated gene (Ndrg1), which encodes a protein that represses downstream the P13K-AKT-mTOR pathway. The authors propose that molecular changes associated with cellular aging may compensate for drivers of age-related decline. Such an approach can be considered potentially useful in individuals who have healthy lungs, i.e., those who have received lung transplantation.

As mitochondrial dysfunction is a major contributing mechanism to skeletal muscle atrophy, exercise, mitochondria-targeted antioxidants, and the use of Peroxisome Proliferator-Activated Receptor Gamma Coactivator 1 via in vivo transfection are potential areas of investigation for therapeutic strategies in skeletal muscle atrophy. Mitochondrial transplantation is another potential consideration for skeletal muscle atrophy in chronic lung disorders [66].

Identifying mechanisms that mediate the adaptive response to exercise has the potential to uncover molecular targets to facilitate the design of new compounds to manage muscle dysfunction and atrophy in chronic respiratory disease. Several signaling, epigenetic, and transcriptional compounds involved in the adaptive response to exercise have been pharmacologically employed in proof-of-concept studies [96,97,98].

## 6. Summary and Conclusions

Skeletal muscle dysfunction, common in patients with COPD, ILD, and post-transplant patients, is systemic and reversible. Patients experience global atrophy and a shift from type I to type II fibers, with decreased capillarization and increased mitochondrial dysfunction. ILD patients experience increased fat infiltration and exercise tolerance while post-lung transplant patients experience increased muscle weakness from prolonged immobility, immunosuppressant toxicity, and metabolic changes. Additional adverse factors include medications (e.g., glucocorticoids), systemic inflammation, hormonal imbalance, and physical inactivity. Atrophy is a consequence of altered balance between protein synthesis and degradation. Technologies grounded in principles of cell biomechanics such as EMG, muscle strength testing, ultrasound elastography, magnetic resonance elastography, along with cellular and molecular biology techniques are utilized to detect and assess muscle atrophy. Integrating clinical diagnostic methods with these tools enhances diagnostic accuracy and treatment efficacy. While isometric tools are typically used for the assessment of limb muscle dysfunction, isokinetic testing better reflects endurance and daily activities.

PR should include a combination of resistance, endurance, and eccentric training to increase strength and quality of life and decrease dyspnea. Programs should be tailored to fit unique patient needs. Nutrition is critical to ensure the patient is adequately fueled. Clinical trials should confirm the benefits of selected supplements.

Avoidance of disease-accelerating environmental factors (such as pollution, stress, sedentary lifestyles, and unhealthy diets), the application of health-promoting lifestyle factors (such as diet, exercise, regular sleeping patterns, and social activities), the administration of relatively non-specific agents with multiple effects (such as MTORC1 inhibitors), or specific medical interventions should contribute to maintenance of muscle, as well as lung health.

## Figures and Tables

**Table 1:** Key studies of skeletal muscle physiology and evaluation of skeletal muscle in respiratory disorders.

Study Name	Year	Authors	Type of Paper	Number of Patients	Methods	Key Findings
**Structural/functional changes of skeletal muscle in chronic lung disease**						
An Official ATS/ERS Statement: Update on Limb Muscle Dysfunction in Chronic Obstructive Pulmonary Disease	2014	Maltais et al. [1]	Expert consensus statement (systematic literature review and update)	Not applicable	A targeted literature review updating the 1999 statement on limb muscle dysfunction in COPD via an interdisciplinary ATS/ERS task force.	Limb muscle dysfunction impairs exercise capacity, reduces quality of life and survival. Review describes atrophy, fiber-type shifts, mitochondrial dysfunction and identifies inflammation, hypoxia, oxidative stress in COPD. In addition to exercise training, neuromuscular electrical stimulation shows promise for early detection and treatment of limb muscle dysfunction.
Relationship between Volitional and Non-Volitional Quadriceps Muscle Endurance in Patients with Chronic Obstructive Pulmonary Disease	2024	Stoffels et al. [3]	Cross-sectional validation study	26 COPD patients (16 completed all tests)	Isometric and isokinetic dynamometry to determine volitional quadriceps activity. Repetitive electrical stimulation to measure non-volitional endurance. Correlations between the outcomes analyzed.	No correlation found between volitional endurance measures and non-volitional force decline. Measures captured different physiological components of quadriceps endurance and were not interchangeable.
Structural alterations of skeletal muscle in chronic obstructive pulmonary disease (COPD)	2014	Mathur et al. [5]	Narrative review	NONE	Review of studies evaluating muscle wasting, structural changes, regenerative capacity, and potential mechanisms for muscle wasting.	About one-third of COPD patients experience muscle mass depletion, which predicts mortality. Key structural changes include smaller type I fibers, lower mitochondrial density, and reduced oxidative capacity. Impaired regeneration implicated by decreased telomere length and central nuclei. Findings possibly related to amino acid metabolism changes, reactive oxygen species, and decreased peroxisome proliferator-activated receptors-gamma-coactivator 1-alpha mRNA. Oxidative deficits show weak functional association while muscle atrophy moderately correlates with function.
Skeletal muscle energetics, acid-base equilibrium, and lactate metabolism in patients with severe hypercapnia and hypoxemia	1987	Fiaccadori et al. [7]	Comparative observational study	10 COPD patients with acute respiratory failure and 10 matched healthy controls	Energy metabolism and acid-base status of the quadriceps muscles were assessed through ATP, PCr, intracellular pH, and lactate levels.	Reduced levels of ATP, PCr, and intracellular acidosis correlated with hypercapnia, and increased muscle lactate. Results indicate anaerobic metabolism and disrupted cellular pathways lead to broader muscle dysfunction despite oxygen supplementation.
Skeletal muscle metabolites and fibre types in patients with advanced chronic obstructive pulmonary disease (COPD), with and without chronic respiratory failure	1990	Jakobsson et al. [8]	Comparative observational study	18 COPD patients (8 with respiratory failure, 10 without)	Quadriceps muscles analysis of ATP, creatine phosphate, creatine, lactate, glycogen, and fiber distribution compared with spirometry and arterial blood gases.	Patients with respiratory failure exhibited lower ATP, glycogen, and creatine phosphate levels. Muscle glycogen correlated strongly with arterial PO_2_. Reduced oxidative type I fibers in both groups indicate metabolic depletion and fiber-type shifts.
Metabolism and aerobic capacity of skeletal muscle in chronic respiratory failure related to chronic obstructive pulmonary disease	1992	Wuyam et al. [9]	Comparative observational study using magnetic resonance spectroscopy	8 COPD patients with chronic respiratory failure and 8 healthy controls	Calf muscle energy metabolism at rest, during incremental exercise, and during recovery assessed using 31P magnetic resonance spectroscopy. Intracellular pH, phosphate (Pi)/phosphocreatine ratio, and phosphocreatine resynthesis rate measured.	Patients showed increased intracellular phosphate (Pi)/phosphocreatine (PCr) ratios, decreased pH, and slower PCr resynthesis. Findings indicate impaired muscle aerobic metabolism, likely due to chronic hypoxemia.
Sex differences in function and structure of the quadriceps muscle in chronic obstructive pulmonary disease patients	2017	Ausín et al. [10]	Cross-sectional comparative study	40 COPD patients (21 women, 19 men) and 15 healthy controls	Different factors including lung and muscle function, exercise capacity, inflammation, fiber type (via quadriceps biopsy), damage-regeneration markers, and inflammatory gene expression were assessed.	More severe muscle dysfunction in women, independent of similar airflow limitation. Women exhibited decreased type II fibers and greater muscle damage while men showed greater regenerative markers. Inflammatory markers elevated in both men and women.
Sex differences in COPD-related quadriceps muscle dysfunction and fibre abnormalities	2019	Sharanya et al. [11]	Cross-sectional comparative study	114 total (76 male and 38 female COPD patients; 30 healthy controls)	Exercise performance, quadriceps muscle strength, and muscle fiber type (via biopsy) compared between male and female COPD patients compared to healthy controls. Plasma cytokines and physical activity also compared.	Female patients expressed decreased quadricep strength, decreased type II fiber CSA, and decreased peak workload compared to men. Higher levels of inflammatory markers, including TNF-α and IL-8 in women, independent of oxygen uptake. Greater susceptibility for muscle weakness and wasting in female patients.
Dehydroepiandrosterone has strong antifibrotic effects and is decreased in idiopathic pulmonary fibrosis	2013	Mendoza-Milla et al. [12]	Case-control and in vitro experimental study	137 IPF patients and 58 healthy controls	Comparisons made between plasma DHEA and (dehydroepiandrosterone sulfate) DHEA-S in IPF patients and controls. Fibroblasts in human lungs exposed to DHEA to determine effect of apoptosis, collagen, synthesis, migration, and proliferation.	IPF patients exhibited significantly lower plasma DHEA and DHEA-S levels. DHEA reduced fibroblast proliferation, increased apoptosis, inhibited TGF-β1-induced collagen production, fibroblast-to-myofibroblast differentiation, and platelet-derived growth factor-driven migration. DHEA deficiency is related to IPF pathogenesis and possesses antifibrotic activity.
Skeletal Muscle Size and Fat Infiltration of the Limb Muscles in Idiopathic Pulmonary Fibrosis (IPF)	2022	Rozenberg et al. [13]	Prospective single-center cross-sectional study	10 male IPF patients	Quadriceps cross-sectional area (CSA) and fat infiltration assessed using B-mode ultrasound and MRI. Functional measures: quadriceps force, 4-m gait speed, six-minute walk test (6MWT), and daily step count.	Ultrasound muscle size strongly correlated with magnetic resonance imaging (MRI). CSA correlated with gait speed, and higher echogenicity correlated with lower 6MWD. Ultrasound is a reliable bedside tool to assess muscle quality and function in IPF.
Skeletal muscle atrophy in advanced interstitial lung disease (ILD)	2015	Mendes et al. [14]	Cross-sectional comparative study	26 advanced ILD patients and 12 healthy controls	B-mode ultrasound used to assess recuts of femoris CSA, and thickness of several limb muscles. Isometric strength testing, short physical performance battery, timed up and go (TUG), and unsupported upper limb exercise tests performed.	Patients showed reduced rectus femoris CSA and limb strength compared to controls. Muscle size was correlated with strength. Advanced ILD is associated with major lower limb weakness and atrophy.
Exercise-induced muscle oxygenation changes in fibrosing interstitial lung diseases: A near-infrared spectroscopy study	2025	Pehlivan et al. [15]	Observational cross-sectional study	36 patients with fibrosing ILD	Changes in muscle oxygenation, total hemoglobin in intercostal and quadriceps muscles assessed using NIRS during a 6MWT.	Intercostal oxygenation higher than quadriceps at rest. Oxygenation stable with exercise, but greater increase in hemoglobin in quadriceps vs. intercostals. Intercostal hemoglobin inversely related to pulmonary function and 6MWT. Suggests blood flow redistribution, not only oxygenation, relates to exercise intolerance in ILD.
Abnormal skeletal muscle oxidative capacity after lung transplantation (LTx) by 31P-MRS	1997	Evans et al. [16]	Observational comparative study using 31phosphorus magnetic resonance spectroscopy (31P-MRS)	9 LTx recipients and 8 healthy controls	Measurements of gas exchange, ventilation, quadriceps pH, lactate, and phosphorylation during incremental quadriceps exercise to exhaustion. 31P-MRS used to assess muscle oxidative capacity.	Reduced peak VO_2_ and normalized lung function in LTx patients. Decreased resting pH and anaerobic metabolic developing earlier during exercise. Exercise limitation rather than ventilatory constraints following lung transplantation correlate with skeletal muscle dysfunction
Skeletal Muscle Oxidative Capacity, Fiber Type, and Metabolites After Lung Transplantation (LTx)	1999	Wang et al. [17]	Observational comparative study	7 lung transplant recipients and 7 age- and sex-matched healthy controls	Peak VO_2_ with arterialized venous sampling during incremental exercise. Quadriceps muscle biopsied to analyze muscle fiber type, metabolite levels, glycolytic and oxidative enzymes, and mitochondrial ATP production at rest.	Patients exhibited decreased muscle oxidative capacity (52% normal VO_2_max). Lower mitochondrial ATP production, oxidative enzyme activity, and fewer type I fibers in biopsy. Phosphofructokinase activity, inosine monophosphate levels, and lactate increased, indicating shift towards anaerobic metabolism. Altered muscle fiber composition and impaired mitochondrial function contribute to exercise limitation following LTx.
31P-NMR study of skeletal muscle metabolism in patients with chronic respiratory impairment	1999	Kutsuzawa et al. [18]	Comparative observational study using 31P-nuclear magnetic resonance spectroscopy (31P-NMR)	9 patients with chronic respiratory impairment and 9 age-matched healthy controls	Forearm muscle analyzed using 31P-NMR spectroscopy during repetitive handgrip exercise. Measurements: intracellular PCr, (PCr)/(PCr + Pi) ratio pH, and PCr recovery time constant.	Impaired oxidative phosphorylation and early activation of anaerobic glycolysis was indicated by a greater decrease in PCr/(PCr + Pi) ratio and pH during exercise. Lung function, muscle mass, and handgrip strength positively correlated with PCr. Skeletal muscle in chronic respiratory impairment reliant on anaerobic energy production and likely related to malnutrition and chronic tissue hypoxia.
Impaired muscle Ca^2+^ and K^+^ regulation contribute to poor exercise performance post-lung transplantation	2003	McKenna et al. [19]	Comparative physiological study	8 lung transplant recipients and 8 healthy controls	Plasma K^+^ and peak VO_2_ measured during incremental exercise. Resting levels of sarcoplasmic reticulum Ca^2+^ release, uptake, and Ca^2+^-ATPase activity, Na^+^/K^+^-ATPase activity and content, muscle pH, buffering capacity, and fiber composition in quadriceps biopsy.	Post-LTx reduced exercise performance related to defective muscle ion transport and excitation–contraction coupling.
Neuromuscular efficiency is impaired during exercise in COPD patients	2021	Frazão et al. [20]	Comparative observational study	COPD patients (mean FEV_1_ = 39.3 ± 13.1% predicted) and matched healthy controls	Surface EMG recorded during cardiopulmonary exercise tests of varying intensities in participants. Respiratory neuromuscular and peripheral efficiency determined by tidal volume to diaphragm to EMG activity and power output to vastus lateralis EMT activity ratio, respectively.	Strong association between reduced respiratory and peripheral efficiency with dynamic hyperinflation, contributing to reduced exercise tolerance.
Neuromuscular and acute symptoms responses to progressive elastic resistance exercise in patients with chronic obstructive pulmonary disease: Cross-sectional study	2021	Calatayud et al. [21]	Cross-sectional experimental study	14 patients with moderate to very severe COPD	Elastic bands of increasing resistance used for participants to perform progressive knee extension. Surface EMG measured muscle activation of rectus femoris, vastus lateralis, and vastus medialis. Vital signs and symptoms recorded.	Quadriceps EMG activity significantly increased after at least two increments of resistance. Cardiorespiratory responses remained unchanged while dyspnea, fatigue, and perceived exertional rate proportionally increased with resistance intensity. Effective method for assessing quadriceps activity with potential for individualized rehabilitation training.
Impact of Chronic Obstructive Pulmonary Disease on Passive Viscoelastic Components of the Musculoarticular System	2021	Valle et al. [22]	Observational comparative study	11 COPD patients and 11 healthy controls	The pendulum test (passive leg oscillations) was used to assess passive knee joint stiffness and viscosity. EMG of the rectus femoris and biceps femoris recorded during voluntary flexion–extension movements.	Significantly reduced stiffness and viscosity during oscillations in patients, showing weaker passive viscoelastic support from connective tissue and muscles. Decreased EMG activity during voluntary motion. Impaired musculoarticular mechanics was a novel contributor to movement limitation in patients.
Thigh Muscle Strength and Endurance in Patients with COPD Compared with Healthy Controls	2006	Janaudis-Ferreira et al. [23]	Comparative observational study	42 COPD patients (26 women, 16 men) and 53 healthy controls	Maximal voluntary contraction (MVC), endurance (number of knee extension repetitions), and FI of thigh muscles were assessed with isokinetic dynamometry. Self-reported levels of physical activity were also collected.	Reduced thigh muscle strength and endurance seen in COPD patients compared to controls, except for knee extension MCV in males. Greater functional decline and higher fatigue index (FI) in females vs. males. Confirms skeletal muscle dysfunction occurring even in physically active patients and there are sex-specific differences that should guide training.
Associations between isokinetic muscle strength, high-level functional performance, and physiological parameters in patients with chronic obstructive pulmonary disease	2012	Butcher et al. [24]	Cross-sectional correlational study	13 patients with COPD	Participants underwent cardiopulmonary exercise testing (aerobic capacity), isokinetic quadriceps strength testing, steep ramp anaerobic test (SRAT), timed up and go (TUG), stair climb power test (SCPT), and 30-s sit-to-stand (STS). Physiological parameters and performance outcomes analyzed.	Strong correlation exhibited between functional performance and anaerobic capacity. No correlation with aerobic power. Strongest determinant of TUG time was isometric peak torque, while the SRAT performance best predicted SCPT. Strongest association with STS was eccentric torque at 90°/sec. High-level functional ability did not depend on aerobic fitness, but rather muscle force generation and power.
Systemic Impairment in Relation to Disease Burden in Patients with Moderate COPD Eligible for a Lifestyle Program (INTERCOM [Interdisciplinary Community-based COPD management] Trial)	2008	Wetering, et al. [25]	Cross-sectional baseline analysis from a clinical trial	127 patients with GOLD stage 2 COPD	Quadriceps, handgrip, and inspiratory force strength, exercise capacity (cycle ergometry, 6MWT), body composition, quality of life (SGRQ, medical research council dyspnea scale) related to hospitalization, smoking status, and exacerbations.	Reduced handgrip, inspiratory muscle strength, and mild quadriceps weakness with 15% showing low fat-free mass. Handgrip strength a predictor of hospitalization risk while diffusing capacity of the lung for carbon monoxide (DLCO) and quadriceps force were independent predictors of exercise capacity. Muscle dysfunction can be detected by early changes in gas transfer.
Quadriceps Muscle Endurance in Patients with Chronic Obstructive Pulmonary Disease	2004	Hul et al. [26]	Observational comparative study	89 COPD patients (57 men, 32 women) and 31 healthy controls (20 men, 11 women)	Quadriceps MVC, endurance (Tlim), dynamic work capacity (Wlim) compared between patients and controls.	Lower strength (MVC), endurance, and work capacity (Tlim, Wlim) exhibited in COPD patients. Only small percentage of differences explained by pulmonary function. Disproportionate reduction in quad endurance compared to strength emphasizes need for PR programs targeting endurance rather than force generation.
Impaired Regenerative Capacity Contributes to Skeletal Muscle Dysfunction in Chronic Obstructive Pulmonary Disease	2022	Jaitovich [27]	Narrative review/mechanistic overview	Not applicable (review-based)	Comprehensive review of current literature.	COPD characterized by decreased mass, contractile strength, fatigue tolerance, and impaired regenerative capacity, related to mitochondrial metabolic dysfunction, affecting muscle repair and satellite cell activation. Potential for exploring mitochondrial–myogenic signaling pathways as therapeutic targets to restore muscle regeneration.
Molecular and Biological Pathways of Skeletal Muscle Dysfunction in Chronic Obstructive Pulmonary Disease	2016	Barreriro et al. [28]	Narrative mechanistic review	Not applicable	Comprehensive review of the current literature.	Discusses multifactorial components relating to dysfunction by describing oxidative stress, inflammation, mitochondrial dysfunction, proteolytic pathway activation (e.g., ubiquitin–proteasome, autophagy), and epigenetic modulation.
Skeletal Myosteatosis Is Associated with Systemic Inflammation and a Loss of Muscle Bioenergetics in Stable COPD	2022	Persson et al. [29]	Observational cross-sectional study	32 patients with stable COPD	Thigh muscle fat infiltration (MFI), and muscle volume was quantified using MRI, muscle bioenergetics (PCr/Pi ratios) assessed via 31P-MRS. Functional, clinical, and biochemical parameters were related.	Thigh muscle composition exhibited increased myosteatosis and reduced fat-free muscle volume. Findings associated with lower physical activity and systemic inflammation. Impaired muscle oxidative metabolism exhibited higher PCr/Pi ratio, lower blood oxygenation, worse airflow obstruction, and greater symptom burden.
Skeletal Muscle Adiposity Is Associated with Physical Activity, Exercise Capacity, and Fibre Shift in COPD	2014	Maddocks et al. [30]	Cross-sectional observational study	101 patients with COPD, 10 healthy controls	Mid-thigh CSA, intramuscular fat percentage, and skeletal muscle attenuation were measured using computed tomography (CT) imaging. These parameters were related to quadriceps fiber-type composition (via biopsy), exercise capacity, lung function, and physical activity.	Lower muscle mass and increased intramuscular fat correlated with reduced physical activity, lower exercise capacity, and a shift from oxidative (type I) to glycolytic fibers. Findings independent of muscle size or strength. When combined with DLCO, imaging biomarkers identified >80% of patients with fiber-type shift. CT provided a non-invasive method to determine muscle composition and phenotype in COPD patients.
Muscle Fibre Type Shifting in the Vastus Lateralis of Patients with COPD Is Associated with Disease Severity: A Systematic Review and Meta-Analysis	2007	Gosker et al. [31]	Systematic review and meta-analysis	Multiple studies (quantitative synthesis across COPD cohorts)	Relationship between fiber-type distribution and COPD severity was collected via analyzing vastus lateralis muscle fiber-type composition and disease severity markers (FEV_1_, FEV_1_/FVC, Body Mass Index (BMI))	Fiber-type shift from oxidative type I fibers to glycolytic type IIx fibers strongly related to disease severity. Positive associations between type 1 fiber proportion and lung function and BMI. Proportions of type I < 27% and type IIx > 29% considered pathologically abnormal. Study established benchmarks for muscle fiber composition and confirmed fiber-type transitions with disease progression.
Cellular Adaptations in the Diaphragm in Chronic Obstructive Pulmonary Disease	1997	Levine et al. [32]	Comparative histologic and biochemical study (biopsy-based)	6 COPD patients, 10 healthy controls	Surgical diaphragm biopsies from patients and controls collected. Immunohistochemistry and electrophoresis used to quantify fiber-type composition and myosin heavy/light chain isoforms.	Shift toward slow-twitch oxidative fibers, with increased slow myosin heavy chain I and decreased fast isoforms IIa and IIb in patient diaphragms. Slow isoforms of tropomyosin, troponin, and myosin light chain upregulated. Reflected fatigue resistance adaptations but increased energy demand from sustained inspiratory load.
Muscle Oxidative Capacity Is Reduced in Both Upper and Lower Limbs in COPD	2020	Adami et al. [33]	Cross-sectional comparative study	20 COPD patients (GOLD 2–4) and 20 smoking controls	Forearm and gastrocnemius oxygen consumption measured by near-infrared spectroscopy as proxy for oxidative capacity. Triaxial (steps/day and vector magnitude units) used to determine physical activity.	Reduced oxidative capacity similar in upper and lower limbs of patients. Findings suggest muscle mitochondrial dysfunction is systemic.
Subcellular Adaptation of the Human Diaphragm in Chronic Obstructive Pulmonary Disease	1999	Orozco-Levi et al. [34]	Comparative biopsy and electron microscopy study	11 COPD patients, 9 controls (undergoing thoracotomy)	Electron microscopy of diaphragm biopsies to evaluate mitochondrial density, sarcomere length, and glycogen content. Findings compared with lung function parameters and maximal inspiratory pressure.	Greater mitochondrial density and shorter sarcomeres in COPD patients, more prominent with air trapping. FEV_1_ inversely related to mitochondrial density and RV/TLC positively correlated with mitochondrial density. Mitochondrial proliferation and sarcomere shortening lead to diaphragm remodeling, enhancing contractile efficiency and endurance.
**Methods of Evaluating Skeletal Muscle Strength and Endurance**						
Body composition, muscle function, and physical performance in fibrotic interstitial lung disease: a prospective cohort study	2019	Guler et al. [35]	Prospective cohort study	115 fibrotic ILD patients (including 40 with IPF)	Handgrip strength, body composition (assessed with Dual energy X-ray absorptiometry), and physical performance (using 4-m gait speed) was assessed. Pulmonary function, dyspnea scores, muscle metrics, and fat metrics collected.	Increased ILD severity related to lower muscle mass, increased fat percentages, reduced grip strength, and decreased gait speed. Age, sex, and weight independent of these factors. Findings most pronounced in men and indicate a strong association with ILD progression, muscle dysfunction, and impaired physical performance.
Relevance of assessing quadriceps endurance in patients with COPD	2004	Coronell et al. [36]	Cross-sectional comparative study	75 participants (COPD patients and age-matched healthy controls)	QMVC and endurance (QTlim—sustained contractions at 10% QMVC until fatigue or 30 min) were measured. Muscle fatigue assessed with EMG and lung function to physical inactivity relationship analyzed.	Quadriceps strength and endurance lower in patients compared to controls. EMG frequency decline occurred in patients, confirming limb fatigue. Lung function, physical activity, or strength levels independent of muscle endurance. Muscle dysfunction not explained solely by reduced airflow or decreased strength.
Isokinetic Testing of Muscle Strength of Older Individuals with Chronic Obstructive Pulmonary Disease: An Integrative Review	2020	Bohannon [37]	Integrative review	27 relevant studies (out of 34 identified)	Search results for “*isokinetic*” and “*chronic obstructive pulmonary disease/COPD*,” using PubMed and a manual literature search. Studies involving isokinetic testing of muscle strength in older individuals with COPD were assessed for validity, reliability, and responsiveness.	Strong evidence for validity of isokinetic strength testing for assessing muscle function in older patients. No evidence provided for supported responsiveness (ability to detect change over time). Isokinetic testing is valid but needs more longitudinal studies.
Reliability of Muscle Strength and Muscle Power Assessments Using Isokinetic Dynamometry in Neuromuscular Diseases (NMDs): A Systematic Review	2022	van der Woude et al. [38]	Systematic review	11 studies across various neuromuscular diseases	Systematic review to evaluate reliability of isokinetic dynamometry for muscle power and strength assessment in patients with NMDs. COSMIN standards used for methodological quality, and disease type used to determine reliability evidence.	Reliable evidence varied among NMDs. High-quality evidence supported reliability best in post-poliomyelitis syndrome compared to other NMDs. Most reliable measure across populations was peak torque. Evidence for chronic lung disease was not reviewed.
Test–Retest Reliability of Lower Limb Isokinetic Endurance in COPD: A Comparison of Angular Velocities	2015	Saey et al. [39]	Reliability study	14 patients with moderate to severe COPD	Patients performed two isokinetic quadriceps endurance tests on two occasions 5–7 days apart. Intraclass correlation coefficient, minimal detectable change, and limits of agreement for torque, endurance, fatigue index, and Borg scale scores used to assess reliability.	High test–retest reliability seen at both velocities for peak torque and total work. Better reliability seen at 90°/s. Dyspnea and leg fatigue ratings were not highly reliable and the fatigue index was only reliable at 90°/s. Isokinetic testing at 90°/s can be useful for assessing quadriceps endurance in COPD.
Associations of the Stair Climb Power Test (SCPT) With Muscle Strength and Functional Performance in People With COPD	2010	Roig et al. [40]	Cross-sectional comparative study	21 COPD patients and 21 healthy controls	SCPT, isokinetic knee torque tests (extensor and flexor), and functional performance measures (Timed Up and Go and 6MWT) were conducted using participants. Associations with these factors and outcomes were analyzed.	Lower SCPT performance and muscle torque in patients compared to controls. Moderate correlation between SCPT and knee extensor torque. Strong correlation between SCPT and 6MWT. The test is a simple, safe, and clinically relevant tool, but is limited by its ability to measure functional power rather than force capacity.
Fixed Handheld Dynamometry Provides Reliable and Valid Values for Quadriceps Isometric Strength in People With COPD: A Multicenter Study	2019	Bui et al. [41]	Prospective multicenter observational reliability and validity study	69 patients with mild–moderate COPD	Measurements of quadriceps isometric maximal voluntary contraction (iMVCquad) using a standardized protocol and relating iMVCquad to functional capacity.	Excellent test–retest reliability with handheld dynamometer (HHD) when compared to computerized standard. No correlation between quadriceps strength measured via HHD and Short Physical Performance Battery (SPPB) performance, suggesting that strength and function assess distinct domains. Fixed HHD is a reliable, valid, and feasible method for assessing quadriceps strength.
The Correlation Between Quadriceps Muscle Strength and Endurance and Exercise Performance in Patients with COPD	2021	Vaes et al. [42]	Large multicenter cross-sectional study	3246 patients with COPD	Peak oxygen uptake (VO_2_) and 6MWT assessed with isokinetic quadriceps strength (QMS) and endurance (QME). Sex and lung function differences taken into account. Regression models and correlations analyzed the relationships between exercise performance and muscle function.	Across sexes and lung function strata, both QMS and QME exhibited high associations with VO_2_max and 6MWT. QME is a stronger determinant of exercise performance. Quadriceps endurance is a better tool to predict exercise capacity than strength alone.
Association between hand grip strength with weaning and intensive care outcomes in COPD patients: A pilot study	2018	Mohamed-Hussein et al. [43]	Prospective observational pilot study	34 COPD patients on mechanical ventilation	Assessed handgrip strength through serial measurements in mechanically ventilated patients. Associations with weaning duration, extubation, successes, and ICU outcomes were compared.	Baseline handgrip strength inversely correlated with mechanical ventilation duration. Day 5 grip strength decreased in reintubated and non-surviving patients. Handgrip strength a potential predictive index for mortality, extubation success, and prognosis.
**Effects of Pulmonary Rehabilitation--Functional and Molecular**						
Skeletal muscle force and functional exercise tolerance before and after lung transplantation: a cohort study	2008	Maury et al. [44]	Prospective cohort study	36 lung transplant recipients (17 male, 19 female)	6MWT, quadriceps force, and lung function determined prior to lung transplantation, measured one month post-transplant, and after completion of outpatient pulmonary rehabilitation (PR). Values related to associations like duration of ICU stay and sex.	Preexisting muscle weakness in patients worsened by ~32% after transplantation, esp. in patients with longer ICU stays. Post-transplant PR significantly increased quadriceps force and 6MWT distance (+140 m). Quadriceps force remained below pre-transplant levels. Females demonstrated decreased functional improvement compared to men.
Associations Between Isokinetic Muscle Strength, High-Level Functional Performance, and Physiological Parameters in Patients with COPD	2012	Butcher. [24]	Cross-sectional correlational study	13 patients with COPD	Participants completed isokinetic dynamometry, Steep Ramp Anaerobic Test (SRAT), TUG, SCPT, and 30-s sit-to-stand (STS). Tests analyzed for anaerobic power, functional outcomes, and muscle strength.	Strong association between muscle strength and anaerobic capacity with functional performance. No association with aerobic power. SRAT performance predicted SCPT, isometric peak torque best predicted TUG, and eccentric torque predicted STS. Findings indicate need for both strength and power training in rehabilitation.
Effects of Whole-Body Exercise Training on Body Composition and Functional Capacity in Normal-Weight Patients with COPD	2004	Franssen et al. [45]	Prospective interventional study	50 COPD patients (plus 36 healthy controls for baseline comparison)	Patients underwent 8 weeks of whole-body inpatient exercise training. Training included daily cycling, treadmill walking, resistance training, and gymnastics. Isokinetic quadriceps strength, exercise capacity (cycle ergometry VO_2_max), and fat-free mass were measured.	Showed increased body weight and fat-free mass and slight increases in fat mass after training. Peak work rate, VO_2_max, and quadriceps strength all improved significantly. Fat-free mass related moderately to VO_2_max and functional gains exceeded changes in muscle mass. Findings highlight functional and anabolic improvements with intensive exercise.
Heavy Resistance Training Increases Muscle Size, Strength, and Physical Function in Elderly Male COPD Patients—A Pilot Study	2004	Kongsgaard et al. [46]	Randomized controlled pilot trial	18 elderly male COPD patients	Two random groups were formed: heavy progressive resistance training twice weekly for 12 weeks compared with conventional breathing exercises. Quads CSA, knee extension and trunk strength, leg extension power, gait speed, stair climb time, chair stands, lung function, and self-reported health assessed pre- and post-interventions.	Significant improvements in quadriceps CSA, isometric and isokinetic strength, leg power, maximal gait speed, stair climb performance, and improved self-reported health in resistance exercise group after 2 weeks. Control group showed no changes. Lung function (FEV_1_) remained unchanged in both groups. Findings show importance of short-term heavy resistance training safely on enhancing muscle size, strength, and functional mobility.
Isotonic Quadriceps Endurance Is Better Associated with Daily Physical Activity Than Quadriceps Strength and Power in COPD: An International Multicentre Cross-Sectional Trial	2021	Frykholm et al. [47]	International multicenter cross-sectional study	81 COPD patients (mean age 67 ± 8 years; mean FEV_1_ 57 ± 19% predicted)	Accelerometry used to assess daily physical activity: steps, sedentary time, and time in moderate-to-vigorous PA (MVPA). Quadriceps strength, power, and endurance (isotonic, isometric, isokinetic) assessed using standardized protocols. Relationships between PA and muscle function were assessed.	Small-to-moderate associations seen between quadriceps function and physical activity. Isotonic endurance was only muscle index shown to improve all physical activity models. When adjusted for isotonic endurance, strength, power, isometric and isokinetic endurance were no longer independently related to physical activity.
Reliability of the Hand-Held Dynamometer (HHD) in Measuring Muscle Strength in People with Interstitial Lung Disease (ILD)	2016	Dowman et al. [48]	Test–retest reliability study	30 ILD patients (10–11 with idiopathic pulmonary fibrosis; mean age ≈ 71–73 years; majority male)	Handheld Dynamometry (HHDy) via two independent raters and two sessions used to measure elbow flexor and knee extensor strength. Inter-rater and intra-rater reliability were analyzed via Bland–Altman plots and intraclass correlation coefficient (ICC).	HHDy exhibited excellent reliability for both inter- and intra-rater testing. Elbow flexors and knee extensors exhibited ICCs of ≥0.95. Mean differences between raters and sessions were minimal, indicating HHDy a reliable method for assessing upper and lower limb muscle strength in ILD patients.
Pulmonary Rehabilitation for Interstitial Lung Disease (Review)	2021	Dowman et al. [49]	Systematic review and meta-analysis of randomized and quasi-randomized controlled trials	675 participants (356 PR, 319 control) across 21 studies	Comprehensive review of PR in ILD conducted via database searches through April 2020. Programs lasting 3–48 weeks were assessed. Primary outcomes (6MWT, dyspnea, and quality of life) and secondary outcomes (peak VO_2_, workload, and ventilation) were evaluated.	Significantly improved exercise capacity with PR. Pooled mean increase in 6MWT = 40 m. Dyspnea decreased and quality of life improved. Gains in 6MWT, dyspnea, and quality of life sustained for 6–12 months post-intervention.
Effects of Pulmonary Rehabilitation in Patients With Idiopathic Pulmonary Fibrosis	2008	Nishiyama et al. [50]	Pilot interventional study	Not specified (pilot study of IPF patients)	A 6-week standardized pulmonary rehabilitation (PR) program was completed by patients with IPF. Functional capacity, fatigue, anxiety, depression, sleep, and health status were assessed before and after PR using validated measures, including the 6MWT and the Fatigue Severity Scale.	Significant improvements in 6MWT (mean 65 m) and a 1.5-point reduction in fatigue severity following PR. Trends indicated better overall health status with reduced anxiety and depression.
Effects of Exercise Intervention on Peripheral Skeletal Muscle in Stable Patients With COPD: A Systematic Review and Meta-Analysis	2021	Li et al. [51]	Systematic review and meta-analysis of randomized controlled trials	1317 participants across 30 RCTs	Five databases searched for randomized control trails evaluating endurance exercise (EE), resistance exercise (RE), and combined exercise (CE) in stable COPD. Skeletal muscle mass, strength, and exercise capacity assessed. Data pooled using standardized mean differences with subgroup analysis by training modality.	Improved strength and exercise capacity seen in COPD patients with exercise. Largest strength gains exhibited with resistance training while endurance and combined exercise increased VO_2_max. Isotonic testing most sensitive measure of strength changes. Resistance training improves aerobic capacity and peripheral muscle function.
Peak Quadriceps Muscle Torque and Electromyographic Output in Patients with Chronic Respiratory Disorders: Effects of Pulmonary Rehabilitation	2023	Grandio et al. [52]	Prospective observational study	18 patients (9 restrictive lung disease, 6 chronic airflow limitation, 3 non-ILD restrictive) and 11 healthy controls	An 8-week pulmonary rehabilitation (PR) program. At baseline, 4 weeks and 8 weeks, isokinetic knee extensions (5 repetitions at 60°/s) performed to record peak quad torque (Tq) and peak EMG signal (Eq) to assess neuromuscular efficiency.	Patients exhibited reduced neuromuscular efficiency at baseline with lower EMG amplitude and lower torque, but a twofold higher Eq/Tq ratio. After 4 weeks there was improved muscle force generation with Eq/Tq decreasing by 44%. No further changes recorded at 8 weeks. Improved St. George’s Respiratory Questionnaire (SGRQ) scores associated with increases in Eq/Tq. PR enhanced quadriceps neuromuscular efficiency within the first month, demonstrating functional adaptation of limb muscles.
Survival After Inpatient or Outpatient Pulmonary Rehabilitation in Patients with Fibrotic Interstitial Lung Disease: A Multicentre Retrospective Cohort Study	2022	Guler et al. [53]	Multicenter retrospective cohort study	701 patients (196 inpatients, 505 outpatients)	Across 12 centers, patients with ILD in inpatient or outpatient PR were assessed with primary outcome (death or lung transplant) in relation to PR.	The 6MWT increased by 55 m (inpatients) and 34 m (outpatients) following PR, which was independently associated with decreased mortality/transplant risk.
Effect of Endurance Versus Resistance Training on Quadriceps Muscle Dysfunction in COPD: A Pilot Study	2016	Iepsen et al. [54]	Randomized controlled pilot trial	30 patients with COPD	Patients randomly assigned to 8 weeks of endurance or resistance training. Assessments included vastus lateralis biopsies (fiber type, enzyme content, capillarization), exercise capacity (6MWT, cycle ergometry), circulatory function, and symptom burden.	Improved exercise capacity and symptoms with both endurance and resistance training. A shift toward oxidative phenotype and reduction of type IIa fibers exhibited by endurance training patients. There were no major metabolic changes with resistance training. Endurance training promoted oxidative remodeling of quadriceps muscle.
Muscle Function and Functional Performance After Pulmonary Rehabilitation in Patients With Chronic Obstructive Pulmonary Disease: A Prospective Observational Study	2022	Pancera et al. [55]	Prospective observational study	20 COPD outpatients	After 5 weeks of PR was completed, assessments collected including 5-repetition sit-to-stand (5STS), 4-m gait speed (4mGS), and measures of quadriceps strength, power, force control, and muscle activation during 5STS. Follow up completed in 3 months.	4mGS and concentric activation during 5STS decreased while quadriceps strength, power, and force control improved with PR and maintained following treatment. During 5STS, eccentric activation showed 31% of 4mGS variance, suggesting its contribution to mobility. Overall, while skeletal muscle properties improved or remained stable, there was a decline in functional performance post-PR, indicating a need for eccentric training to enhance long term outcomes in rehabilitation.

## Data Availability

No new data were created or analyzed in this study.

## Abbreviations

The following abbreviations are used in this manuscript:

1STS	One-Minute Sit-to-Stand
4mGS	4-Meter Gait Speed
5STS	Five-Repetition Sit-to-Stand
6MWT	Six-Minute Walk Test
ARFI	Acoustic Radiation Force Impulse
ATP	Adenosine Triphosphate
BMP	Bone Morphogenetic Protein
Ca^2+^	Calcium Ion
CINAHL	Cumulative Index to Nursing and Allied Health Literature
CO	Cardiac Output
COPD	Chronic Obstructive Pulmonary Disease
COSMINQMS	Consensus-Based Standards for the Selection of Health Measurement Instruments Quality Management System
CSA	Cross-Sectional Area
CT	Computed Tomography
CTD-ILD	Connective Tissue Disease-Related Interstitial Lung Disease
CV	Conduction Velocity
DHEA	Dehydroepiandrosterone
DHEA-S	Dehydroepiandrosterone Sulfate
DLCO	Diffusing Capacity for Carbon Monoxide
EFL	Expiratory Flow Limitation
EMG	Electromyography
Eq/Tq	Quadriceps Torque-to-EMG Ratio
FEV	Forced Expiratory Volume
FI	Fatigue Index
FVC	Forced Vital Capacity
HHD	Handheld Dynamometer
HIF-1	Hypoxia-Inducible Factor 1
HP	Hypersensitivity Pneumonitis
HR	Heart Rate
ICC	Intraclass Correlation Coefficient
ICU	Intensive Care Unit
ILD	Interstitial Lung Disease
IL-6	Interleukin-6
IL-8	Interleukin-8
INTERCOM	Interdisciplinary Community-Based COPD Management
K^+^	Potassium Ion
K921	Myocyte Lysine 921
MEP	Maximal Expiratory Pressure
MES	Surface Myoelectric Signal
MF	Median Frequency
MID	Minimal Important Difference
MFCV	Muscle Fiber Conduction Velocity
MIP	Maximal Inspiratory Pressure
MRI	Magnetic Resonance Imaging
mTORC1	Mammalian Target of Rapamycin Complex 1
MVC	Maximal Voluntary Contraction
MVIC	Maximal Voluntary Isometric Contraction
MVPA	Moderate-to-Vigorous Physical Activity
ncRNA	Non-Coding Ribonucleic Acid
NF-κB	Nuclear Factor-Kappa B
NIRS	Near-Infrared Spectroscopy
NMD	Neuromuscular Dysfunction
NMR	Nuclear Magnetic Resonance
PA	Physical Activity
PCr	Phosphocreatine
PR	Pulmonary Rehabilitation
QME	Quadriceps Muscle Endurance
QMVC	Quadriceps Maximal Voluntary Contraction
RMS	Root Mean Square
ROS	Reactive Oxygen Species
SCPT	Stair Climb Power Test
SCE	Spectral Compression Estimate
SE	Strain Elastography
SGRQ	St. George’s Respiratory Questionnaire
SPPB	Short Physical Performance Battery
SRAT	Steep Ramp Anaerobic Test
STS	Sit-to-Stand
SWE	Shear Wave Elastography
TGF-β	Transforming Growth Factor-Beta
TLC	Total Lung Capacity
TNF-α	Tumor Necrosis Factor-Alpha
Tq	Peak Torque
TUG	Timed Up and Go
UE	Ultrasound Elastography
VO_2_max	Maximal Oxygen Consumption
